# Prevention of sexual abuse: improved information is crucial

**DOI:** 10.1186/1753-2000-8-5

**Published:** 2014-02-12

**Authors:** Hans Zollner, Katharina A Fuchs, Jörg M Fegert

**Affiliations:** 1 Istituto di Psicologia – Centre for Child Protection, Pontificia Università Gregoriana, Rome, Italy; 2 Klinik für Kinder- und Jugendpsychiatrie und Psychotherapie, Universitätsklinikum Ulm, Ulm, Germany

**Keywords:** Prevention, Sexual abuse, Effectiveness, Evaluation

## Abstract

Sexual abuse can lead to long-lasting, even life-long, consequences and is a serious problem on an individual, familial and societal level. Therefore, prevention measures on different levels are a public health issue. Minors as well as adults should be involved in prevention work in order to prevent sexual abuse of minors in a sustainable way. Besides norms, structures and values in society, the respective laws as well as attitudes and structures should be changed and amended in such a way that abusers and the abuse are clearly confronted everywhere. In the last decades, numerous prevention programs for victims have been developed for various target groups (e.g. parenting education classes, home-visiting programs, public education, training sessions for teachers, E-Learning Programs of the German Federal Ministry for Education and Research and the Centre for Child Protection). Many of these programs have proven partially effective. Nevertheless, until now there is no consensus in the scientific community on what constitutes effectiveness in this context. Reasons for this are the discrepancies in definitions or the scarcity of attention which the evaluation of prevention measures has received.

## Background

During the Round Table on “Childhood Sexual Abuse” organized between 2010 and 2012 by three German Federal Ministries (the Ministry for Research and Education, the Ministry for Families, Senior Citizens, Women and Youth and the Ministry of Justice) one of the key questions revolved around ways in which the renewed large-scale victimization of children and youth in institutions, but also in families could be avoided in the future. Furthermore, Dr. Christine Bergmann, a former Federal Minister was appointed Independent Commissioner to investigate the sexual abuse of children. Many of those affected who had turned to the Independent Commissioner for Childhood Sexual Abuse [1], had requested generally better prevention and earlier intervention to avoid potential harmful consequences for the abused. Several areas were identified as central to the dissemination of improved information and prevention strategies, including specific psychotherapy, school and leisure education as well as volunteer activities associated with the church or sports. The need for continued education seems to be particularly high in institutions which are taking care of a population that is already more endangered, such as children’s homes and boarding schools. The study ordered by the Independent Commissioner showed that particularly in this latter area, directors of such homes and schools saw a clearly increased risk in being confronted with such scenarios [2]. The German Federal Ministry for Families, Seniors, Women and Youth charged the German Association for the Prevention and Intervention (DGfPI) into Childhood Abuse and Neglect with the development of a continued education program for institutions providing youth services. Currently, these programs are being established nation-wide with experts from youth counselling services. To improve the information available to health and education professionals, the Federal Ministry for Research and Education has financed the development of an e-learning program [3]. The Pontifical Gregorian University in Rome in 2012 founded the Centre for Child Protection (CCP) and tasked it with developing an internationally-oriented e-learning program for use by the Church [4]. To do so, a consortium was established in Munich, Germany, with the support of the Arch Diocese of Munich-Freising and other sponsors. During the initial phase, the consortium will be able to draw on the expertise of the Medical Faculty in Ulm, Germany, in the areas of child protection and e-learning. During the Round Table discussions, there was agreement that the rapidly implemented activities to improve victim-focused prevention measures needed to be evaluated. However, there was not much discussion about the criteria that would be used to measure success, beyond simply indicating satisfaction. This is largely due to the fact that there are differing ways to conceptualize and implement prevention. This article is an attempt to integrate specific prevention activities for childhood sexual abuse into the broader public health debate on prevention. The focus in a public health debate on prevention should lay on sensitive and carrying environments and the responsibility of adults instead of putting too much responsibility on the child to learn to protect himself/herself from sexual abuse. In about 80% of the abuse cases the perpetrators are persons known to the child and his/her family [5,6]. Therefore, the adult environment of a child should be a main focus of prevention approaches. The article will point out corresponding challenges in prevention strategies and highlight potential approaches to evaluation.

### How to define prevention?

**Figure 1:**
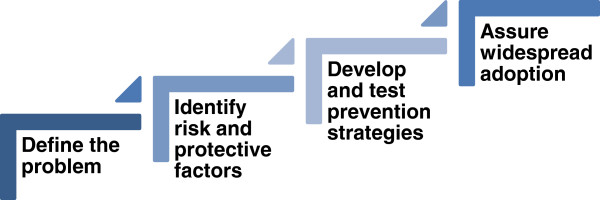
**Public Health Model (according to Center for Disease Control and Prevention (CDC)**[9]**).**

There is no uniform, generally applicable definition of prevention. However, one can point to a few commonalities among the various definitions. For Bloom [7], for instance, prevention is usually interdisciplinary and has as its goal a long-term change on several levels. Conceived more broadly, prevention can be understood as a path to reducing potential injuries to body and soul and, simultaneously, to encouraging positive interactions and contexts. With regard to sexual violence, prevention means creating healthy and safe circumstances and behaviors so as to prevent sexual crimes before they can even take place [8]. Prevention of sexual violence is not easy to measure which makes a critical discussion as well as regular evaluation of prevention work and prevention approaches all the more indispensable [8]. The *Public Health Model* for the prevention of violence, adapted from the US Center for Disease Control and Prevention, understands prevention mainly as a reduction of risk factors and an increase in protective factors with the goal to prevent sexual violence from happening in the first place [9]. The *Model* (see Figure 1) spells out four levels: [1] defining the problem, [2] identifying risk factors and protective factors, [3] development and testing of prevention strategies, and [4] assuring widespread adoption of these strategies.

Preventive approaches to sexual violence can take many forms. Victim-focused prevention, for instance, can include *relationship-based prevention* (changes to and/or control over potentially dangerous relationships through political or legal measures) and/or *behavior-based prevention* (support for developing active and self-protective modes of behavior and strategies) [10].

With regard to behavior-based prevention, the following differentiations can be made:

● Primary prevention

● Secondary prevention

● Tertiary prevention

This concept [11] was adopted by the WHO in 1994 and is often used in curricular prevention programs. In this context, *primary prevention* refers to each and every measure that is taken to reduce sexual violence from the outset, for instance through workshops with minors. *Secondary prevention* includes measures used in high-risk situations, for example in order to put a stop to abuse that is emerging or in progress. The reaction to sexual crimes already committed is the domain of *tertiary prevention* which aims at mitigating the immediate consequences of abuse and at minimizing secondary consequences. Additionally, this level comprises relevant therapies and measures to prevent a relapse in order to strengthen the psychological and physical health of those affected, cf. among others [12]. So far, a majority of current political measures for the prevention of sexual abuse rests on secondary and tertiary prevention. Primary prevention strategies have received scant attention in prevention programs as well as in internationally-oriented prevention policies.

Besides the distinctions in Caplan’s [11] “classical” concept described above, Gordon’s [13] more person-oriented concept of prevention, introduced by the *Institute of Medicine* (IOM), has been increasingly been employed. Gordon’s model differentiates between *universal, selective and indicated* prevention, cf. [12,14,15]. Unlike the previous model, Gordon [13] does not make a distinction with regard to the time-line of measures but rather with regard to the target groups which are to be reached by the measures. *Universal prevention* is directed at the overall population of a country, a region, a city or a company’s employees or a school’s students. *Selective prevention* is geared towards a group or individuals who are at higher risk for abuse based on specific, generally well-known conditions. *Indicated prevention* takes place when individuals find themselves in risk-prone situations or when they exhibit unusual behavior (such as a sudden drop in school grades to which parents and teachers react quickly and attentively).

**Figure 2:**
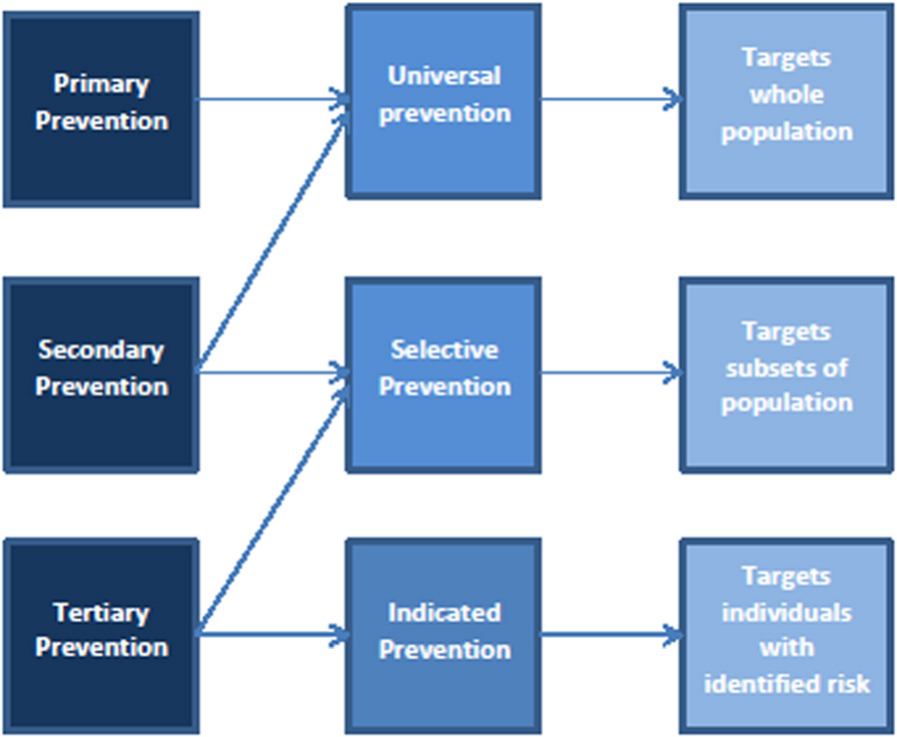
**Overlap between the types of prevention (according to Meili, **[17]**).**

Figure 2 illustrates the fact that these two concepts [11,16] cannot only be used separately but also in combination and through integrated approaches.

As named above, secondary prevention includes measures used in high-risk situations, therefore it is, as Meili [17] suggests, not only important for individuals at a high risk for abuse but for example also for the overall population of a certain region, city or school, where the individuals are at a high risk. Furthermore tertiary prevention can not only be seen as indicated prevention; tertiary prevention can, according to Meili [17], be understood as selective prevention by trying to minimize the immediate and the secondary consequences for a group or individuals at a high risk for abuse.

**Figure 3:**
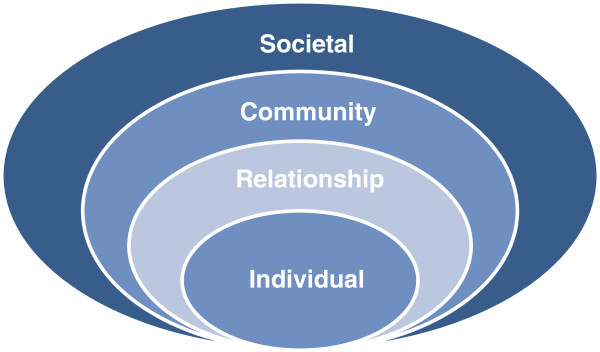
**The Social-Ecological Model (according to Dahlberg & Krug, **[19]**).**

In their *Spectrum of Prevention Model*, Cohen and Swift [18] point to the importance of an organizational approach. According to their model, the prevention of sexual abuse occurs on several levels: from the individual to the organizational level. They argue that in order to work towards prevention of sexual abuse, political goals and legal frameworks also have to undergo a change on the organizational level. This concept corresponds to the approach of the *Social-Ecological Model* by Dahlberg & Krug [19] which identifies four levels of a person’s social environment as necessary for abuse prevention (cf. Figure 3): the individual level, the relationship level, the community level and the societal level.

In order to prevent sexualized violence against minors and sexual abuse sustainably and over the long term, prevention has to address several levels. Often-cited reasons for worldwide sexual violence are: the unequal treatment of the sexes, economic disparities as well as social and cultural norms which sanction particular images of masculinity predicated on the control of women, and which value male strength and toughness [20]. In public debates and political discussions, however, this analysis is only rarely linked to the sexual abuse of minors. The marked increase in the national [2] and international [21] public’s and media’s interest in issues of sexual abuse have nevertheless led to increased efforts by more and more nations (such as Germany, England, South Africa, Australia or the Philippines) which are attempting to actively pursue policies against sexual abuse [22-24]. Examples include training measures in schools to recognize the signs of sexual abuse in minors, or, with regard to employment, checking official records for indications of previous relevant arrest records. As Finkelhor [16] has been able to show, prevention work against childhood sexual abuse has become widespread in schools internationally and is frequently supported politically.

Before we address prevention and prevention programs in detail, a brief terminological clarification may be necessary, namely the distinction between *efficacy and effectiveness.*

A treatment proves *effective*, if it works under non-ideal conditions in everyday practice [25]. Scientists, clinicians and politicians often are called upon to differentiate between the *effectiveness* and the *efficacy* of a prevention or intervention. *Efficacy trials* (explanatory studies) state whether prevention or intervention measures produce the expected result under quasi experimental conditions. *Effectiveness trials* (pragmatic studies), on the other hand, measure the extent of the positive effectiveness under “real-world” conditions in everyday settings. For this reason, researchers formulate hypotheses and study designs of *effectiveness trials* based on certain conditions, such as routine clinical practices and the importance of the trial’s results for clinical decisions. *Effectiveness* cannot be measured through the use of controlled, experimental studies, as the simple fact of inclusion in a study leads to distortion of customary practice. *Effectiveness* can be assessed via observational studies in the actual field and allows for both qualitative as well as quantitative assessments.

*Efficacy* and *effectiveness* exist on a continuum [25,26]. Generalization depends on the perspective of the researcher or observer as well as on the conditions prevailing during the study. The patients’ basic data (e.g., gender, age, severity of illness, racial groups) are vital factors for the generalisability; this means that the generalization of one and the same study can, depending on the population, vary from low to high [25].

### Effectiveness of prevention in general

Effective prevention strategies operate on various levels: on the level of minors; on the level of adults who live or work with children and youth; but also on the level of societal norms and values, on the legislative level, and on the level of attitudes and structures which – more or less intentionally and more or less “consciously” – protect perpetrators or downplay their behavior (cf. E-Learning Program of the German Federal Ministry for Education and Research and the Centre for Child Protection [3,4]).

Two aspects deserve particular attention for successful prevention work with regard to childhood sexual abuse: content areas (What does one have to know?) and structures (Which methods are being used? What kind of institutional and personal assistance is being offered?). The content areas of prevention measures and programs determine to a large degree how effective the changes in the behavior of minors and adults are [27]. The structure decisively influences whether the program or measure is effective in the long term [27]. Victim-focused prevention of sexual abuse of minors is based on competent and comprehensive information and has as its goal the effective protection of children. The focus is on conveying factual and comprehensive information which takes into account the specific circumstances, needs and resources of the person and his/her environment. In addition, victim-focused prevention highlights various prevention options and integrates co-operating partners on various levels [12,27]. Besides conveying content, changes in structural components are decisive for successful prevention work. As several survey studies by Knorth, Knot-Dickscheit & Strijker [28] have shown, there exist identifiable structural factors which are highly promising. These include: providing solid basic information; using a variety of prevention methods; involving parents, family members, teachers, peers or other contacts; providing a precise and competent introduction to a prevention program or a specific prevention measure and its consistent implementation in the respective context (e.g., school, sports club, parish) [28]. Additional factors which determine the success of a given measure, include materials used and their mode of integration into the program; the didactic implementation and length of the measure; the professional qualifications of those implementing the measure and those in positions of responsibility; implementation that takes into account gender and culture-specific aspects. Moreover, studies indicate the effectiveness of close interdisciplinary networks and cooperation with counselling and therapeutic services, trauma ambulances, emergency hotlines, abuse commissioners, ombuds posts and/or the police and respective courts [27].

The following aspects are – as shown in existing studies for an overview see [27,28] – the hallmarks of a successful prevention program:

–  Prevention measures are directed primarily at adults and only secondarily at children and youth; this puts the responsibility for the protection of minors from sexual abuse squarely in the hands of adults.

–  Prevention measures are implemented in frequent, short, and regular intervals.

–  Prevention measures employ appropriate language; it is important to provide compact information that is easily understandable, specific and comprehensive and which does not ask too much of the target group.

–  In the case of children, relevant questions include if and to what extent they have had sex education.

–  Both girls and boys are equally and equivalently seen as potential victims.

Prevention programs are implemented by a team representing both genders.

–  Prevention measures confront the day-to-day complexities of a specific target group; this means that besides gender and language, culture, religion, politics, status as well as the legal system of the respective state is taken into account.

–  Many prevention programs (e.g., including the E-Learning-Program of the German Federal Ministry for Education and Research as well as the *Centre for Child Protection* at the Pontifical Gregorian University in Rome) offer basic information on potential intervention measures.

For ethical reasons, it is often difficult to study evidenced-based prevention in the field, particularly with regard to sexual abuse [29]. This is probably one of the reasons why there are few existing comprehensive studies which would allow to make statements about the empirical basis for the efficacy of prevention. Additionally, there are hardly any studies which make valid statements about the efficacy and the long-term effectiveness of prevention measures [27].

### Types of prevention programs and their effectiveness

Over the course of time, numerous prevention programs for the protection of minors from sexual abuse have been developed. These programs show that in most cases it is not sufficient to educate minors in order to prevent sexual abuse. Truly effective *primary* prevention not only means that everything is being done to minimize the number of sexual criminal offenses; it also includes broadly disseminating information to the general public and corresponding actions. While public awareness of childhood sexual abuse in the society at large and also in Church contexts has increased significantly over the past few years in North America and in Western and Central Europe, many societal groups, countries, and cultures are still lacking information on the issue, especially with regard to prevention. The task then consists in not only providing information but also in developing prevention strategies and programs, in implementing them and in evaluating their effectiveness within the respective cultures and contexts. Prevention programs are directed either primarily to minors or to adults.

### Prevention measures primarily addressed to minors

In order to be able to protect themselves from unwanted advances by adults, minors need to be able to recognize inappropriate behavior as such and to respond to it accordingly. Therefore, a policy mix of behavioral prevention and structural prevention is necessary. Prevention programs which are specifically geared towards minors most often take the *empowerment* approach. Of central concern in this approach is acquiring skills for self protection which are conveyed via concepts and practical exercises, such as saying no, avoidance, running off and reporting. These four skills are at the center of training programs for the prevention of sexual abuse and have proven effective [30]. Minors themselves have perceived and evaluated them as positive, cf. [31]. Through these training programs, they learn to *recognize* situations which endanger their personal safety, to *avoid* similar situations, to *escape* the danger and afterwards to immediately *tell* a trusted adult (behavioral prevention), but on the base of a long-term effectiveness it is also important to know which adults are trustworthy and to have such adults available (structural prevention). These adults might be for example school-psychologists or liaison teachers in schools.

In the past, researchers have primarily used three types of assessment to evaluate the effectiveness of safety training for children and youth: verbal reports and/or self-reporting; role plays and construction of realistic cases (*in situ)*. Research has proven that an evaluation *in situ* is the only valid assessment criterion with regard to skills that increase personal safety. These results were confirmed in the case of women with mental retardation who received training in prevention strategies against sexual abuse, e.g., [32]. According to Knorth et al. [28], meta-analyses and review studies show positive effects of school-based prevention programs merely with regard to general protection factors, but not with regard to prevention of sexual abuse. In assessing the effectiveness of prevention programs, researchers need to critically evaluate what minors themselves say about a particular measure and/or their increased sense of personal safety. Important questions in this context include: To what extent are minors able to implement the skills and strategies practiced in the event of an emergency? How high is the risk of traumatization through role-playing of attempted advances? Obviously, children who defend themselves are merely able to delay the perpetration but not to avoid it, cf. [31]. Part of the reason for this seems to stem from the fact that prevention programs geared at children and youth often neglect those aspects which allow for sexual abuse to occur. Areas and circumstances over which minors have no or only slight control and which fall under the complete or primary control of adults, are neglected [31]. This includes intercultural and interreligious contexts to which the majority of existing programs do not pay attention, because they orient themselves on Western cultures and their norms and regulations [27]. Moreover, it needs to be critically remarked that many *primary prevention* measures which focus on the acquisition of self-protection skills delegate the responsibility for the protection from abuse to potential victims, i.e., to minors. These minors, then, carry the burden of a responsibility which they are generally unable to shoulder. The responsibility for the protection of minors lies with adults. It is indispensable for effective and successful prevention that minors not only acquire the skills but that they learn to apply them in a dangerous and high-risk situation by avoiding or escaping from it [27].

### Prevention measures additionally or exclusively addressed to adults

Some prevention programs are addressed exclusively to minors, others involve adults as well [33], still others are geared exclusively to adults. Recent studies show that the involvement of adults in the prevention of child sexual abuse is important. Through their involvement, adults not only learn how to talk with minors about sexuality and their sexual and emotional development but also how to recognize problematic behavior, how other adults can be held accountable for their wrong behavior, and what to do when signs of sexual abuse exist [34]. As the US-based National Sexual Violence Resource Center (NSVRC) was able to show in 2005, the inclusion of adults in prevention work brings with it many advantages [35]. For instance, minors are better able to grasp risk-reducing messages, when these are conveyed by adults at home or in school. In addition, care providers, teachers, coaches, or educators should receive advice and information about where to ask for assistance and support in emergencies.

Table 1 provides an overview of various types of prevention programs addressed to various groups of adults as well as the programs’ goals and effectiveness.

**Table 1:** Overview of various types of prevention programs addressed to various groups of adults

	**Parents/Guardians**	**People working with children (e.g., teachers, coaches)**	**General public**
**Goals**	To educate parents and guardians on.....,	To educate people working with children on....,	
	1. How to inform their children about sexual abuse	1. How to inform children about the prevention of sexual abuse	1. To inform the public about sexual abuse (e.g., prevalence, opportunities for intervention, etc.)
	2. How to protect their children from sexual abuse	2. How to recognize and report sexual abuse	2. To change societal behavior
	3. How to recognize signs of (potential) sexual abuse und how to stop it		
	4. How to strengthen a healthy family dynamic		
**Challenges for the conceptualization and implementation of effective measures**	Important factors for the success of a program addressed to parents/guardians are:	Important factors for the success of a program addressed to those working with children are:	
	• Interest in the topic	• Interest in the topic	• Media-based campaigns are complex in content and costly to finance
	• Qualification of the prevention educator	• Qualification of the prevention educator	
	• Program time-frame (length of training measures and/or independent work)	• Program time-frame (length of training measures and/or independent work)	• Their effectiveness can indirectly depend on the availability of monetary donations
	• Source of referral (Parents put more trust into recommendations by physicians or teachers than those by the media)	• Source of referral (similar to those of parents/guardians)	• Social-marketing campaigns need to be based on solid research with a view towards the target group
**Studies to evaluate prevention programs**	• Parents who have participated in a program led by qualified educators and have discussed the topic, are in a stronger position to protect a child from sexual abuse [36]	• Programs addressed to teachers have a positive influence on the teachers’ knowledge about the topic [37]	• Up to now, only very few public campaigns on this topic have been assessed [34]
			• Campaigns that focus on specific target groups and topics receive more attention and are therefore more effective [34]
	• Programs which include both parents have consistently proven to be effective [37]. “Home visitations “in high-risk families result in a decrease in the risk of abuse [38]	• People who received training feel in a much stronger position to make a decision not solely based on physical signs; in addition, frequency, duration, intensity, and the professional standards of training measures play an important role [39]	

Legend: Overview of goals, of challenges for the conception and implementation of effective measures as well as of studies on the evaluation of prevention programs for adults.

A further central aspect in prevention work with adults is *bystander education*, which aims at sensitizing the largest number of people possible to the topic. The principle of *bystander education* focuses on expanding the prevention of abuse from the individual and familial level to the societal level. This means that members of specific groups of society but also the general public as a whole are encouraged and strengthened to work against sexual and other forms of violence [22]. Such an approach includes the questioning of prevailing social norms such as the private sphere, power, gender roles, etc. [40]. As Cohen, Lyles & Brown [41], for instance, were able to show, *bystander education* has a positive effect. Some prevention approaches in this context attempt to react to clearly identifiable shortcomings (for instance, when teachers do not know how to report a suspicion, they receive training on this particular aspect). Other approaches are oriented on *common sense* (“We have to keep children away from sex crime offenders.”); still others result in laws, which, for example, proscribe where such offenders are allowed to live.

## Discussion

For a number of years now, research has been undertaken into the evaluation of prevention programs for sexual abuse, e.g., [30,42]. Numerous studies point to the fact that the relevant knowledge about sexual abuse and the necessity for increased prevention has been on the increase, e.g., [43]. This finding could be confirmed for safety training programs with children, especially when these training programs included an active approach to learning, e.g., [44,45]. The significance of these studies is limited as there is usually no behavioral assessment but only self-assessment on the part of the children. This means that researchers and program educators have to rely on these self-assessment without being able to observe the behavior of minors *in situ*[46]. Studies, e.g., [44], which have instituted *in-situ assessments* as basis for evaluation, have shown that minors behave differently in situations which could lead to potential abuse than they stated previously. Willich [47] and Damrow [27] comment further that the evaluation of the effectiveness of prevention programs for minors often contains qualitative flaws which, in turn, limits their significance. Examples for such flaws are: the use of divergent assessment criteria for the evaluation of preventive measures, a scarcity of studies with regard to cost effectiveness of measures taken as well as a lack of longitudinal studies, e.g., [27]. It is only through cooperation, regular evaluation and the continuous work of many that prevention programs can be further developed in the future to educate and encourage adults to protect minors from sexual abuse [34].

In the meantime, many programs addressed to adults have been evaluated according to strict empirical criteria. Table 1 provides a detailed survey. A considerable number of diverse prevention measures with strong financial support focus on adults as the target group. These programs were evaluated and implemented in specific societal contexts. These local efforts work towards a culturally competent approach which focuses on the specific needs of divergent societal groups which are often neglected or overlooked in broader campaigns [34]. Increased cooperation among institutions can benefit the development of ideas and resources along these lines.

Let there exist different definitions of level of behavioral prevention which have to be considered and structural preventive measures. E.g., in substance abuse prevention structural measures like nationwide taxation policies prove to be very effective and at the same time easy to evaluate. Still it seems difficult to describe pure structural measures of prevention in the context of child sexual abuse because the structures surrounding a child in the sense of a sensitive environment are created by behaviors and emotional availability of teachers and other caregivers or peers. As a structural measures creation of networks, the installation of an ombudsman could be mentioned but there is not much empirical literature about structural measures in the prevention of sexual abuse. Rassenhofer et al. [23] reported about a critical incident reporting system established by the German Federal Government with a government commissioner, functioning as an ombuds person. This example shows that a trustworthy adult person is essential for effective prevention and early intervention. If the child does not know who to tell about and how to find an adult that will listen, believe and help, behavioral trainings for children will not beware them from sexual abuse for a long time. So it is necessary to sensitize adults and to aside a qualified, trustworthy person to the child.

### The political contribution

Adequate evaluation of prevention programs has to face numerous hurdles: scarcity of finances; lack of professional expertise; additional time requirements; society’s critical attitude toward research findings. In some cases, politicians, lawyers and scientists felt under pressure to “have to” confirm the effectiveness of a particular program. Such pressure can lead to distorted data analysis and interpretation of research results [48]. For this reason, researchers [49,50] plead for a broader understanding of effectiveness as well as for a more integrative evaluation strategy which combines various approaches. According to Tseng [50], such an approach could prevent the neglect of promising strategies and promote new approaches to find solutions for urgent societal questions. In the medium and long-term, an integrative approach to knowledge generation will be helpful in better protecting minors and families in danger. In addition, the use of multiple evaluation methods allows for a more comprehensive assessment of the effectiveness of prevention programs [49]. Proceeding from these findings, further steps for prevention work in organizations and institutions can be deduced [2,51]. Partnerships and cooperative projects between politicians and researchers could give rise to political prevention measures whose societal effect and preventive effectiveness will continuously have to be re-assessed [2,23,50]. Groups, institutions, and organizations as a whole as well as those who hold positions of responsibility within them are called upon to regularly evaluate the effectiveness of their political work.

## Conclusions

The present article provides a survey of prevention concepts in general and of prevention programs and political measures against sexual abuse of minors in particular. Furthermore, it highlights the difficulties in the evaluation of the effectiveness of preventive measures to protect minors from sexual abuse. A central point is the development of targeted evaluation methods and/or the review and critical assessment of existing strategies. Programs to avoid sexual abuse have to be regularly and validly reviewed for their effectiveness and, if necessary, be modified within a particular context or, if they result to be in-effective, discarded.

So far, there exists a multitude of different prevention programs which address a different clientele (minors and/or adults), work with a variety of methods (e.g., e-learning, face-to-face) or are targeted towards different contexts (school, family, clubs, church, etc.). Besides these, there are a number of efforts on the political (e.g., the Independent Commissioner for the Investigation of Child Sexual Abuse) [1,2] and church level (e.g., e-learning program of the German Federal Ministry for Education and Research, Centre for Child Protection) ([52], Zollner H, Fuchs KA: **Wirksamkeit von Prävention,** forthcoming) as well as public campaigns. Many of these strategies and programs intend to protect children and youth effectively from sexual abuse [51]. However, too much euphoria may be unwarranted. Quantity does not automatically also mean quality and what seems to be effective at first glance, may not be so after empirical review. This means that prevention programs against sexual abuse need to be evaluated conscientiously and regularly with reliable methods and need to be developed further [23]. From today’s vantage point, no one can predict which type of prevention or which combination of different strategies will be most effective. Many programs have to be adapted to relevant specific contexts and needs.

## Competing interests

HZ gets funding from the Archdiocese of Munich and the Gregorian University for the development of E-Learning programs for the prevention of sexual abuse.

## Authors’ contributions

HZ, KF, JF contributed equally to the preparation of the article. All authors read and approved the final manuscript.

## Authors’ information

JMF: Prof. Dr. med.; Professor and Chair of Child and Adolescent Psychiatry and Psychotherapy at the University of Ulm/Germany. Direction of the Scientific Working Group of the “Round Table on Child Abuse” by the Federal Government of Germany. Member of the Steering Committee of the “Centre for Child Protection” of the Pontificial Gregorian University.

HZ: Prof. Dr. theol. Lic. psych.; Academic Vice-Rector of the Pontificial Gregorian University in Rome/Italy. Dean of the Institute of Psychology and President of the “Centre for Child Protection” of the Pontifical Gregorian University.

KAF: Dott.ssa; Dipl. Psych.(Univ.); Lecturer at the Institute of Psychology of the Pontifical Gregorian University in Rome/Italy. Research Staff Member at the “Centre for Child Protection” of the Pontifical Gregorian University.
